# News and Views (3 & 4): JPS Young Scientist Award, 47th AAPPS Council Meeting

**DOI:** 10.1007/s43673-022-00034-7

**Published:** 2022-04-13

**Authors:** AAPPS Bulletin

**Affiliations:** Association of Asia Pacific Physical Societies, Pohang-si, South Korea

## Young Scientist Award of the Physical Society of Japan, 2022 by JPS

Every year, the Physical Society of Japan (JPS) presents Young Scientist Awards to young researchers who have made outstanding achievements in their early research careers. This year’s winners were recently decided by the board of directors of JPS according to the recommendations from the selection committees established in 19 divisions of JPS. The maximum number of winners from each division has been determined based on the number of talks given at the Annual Meetings in the past three years. All the winners are expected to give an award lecture at the next Annual Meeting of JPS, which is scheduled for March 2022. Here is the list of winners and their respective research topics.

### Theoretical Particle Physics

Masamichi Miyaji (University of California, Berkely)

“Proposal regarding surface/state correspondence”

Yoshiki Sato (National Center for Theoretical Science, Physics Division)

“Studies on the defect C-theorem”

Ryo Nagai (Osaka University)

“Generalized Higgs effective field theory and perturbative unitarity”

### Experimental Particle Physics

Yohei Noguchi (Department of Physics, Graduate School of Science, Kyoto University)

“Measurement of Higgs boson properties using the decay channel to b-quarks following associated production with a vector boson in pp collisions at √s = 13 TeV”

Ayami Hiramoto (Okayama University)

“Measurement of neutrino interactions on water using nuclear emulsion detectors”

Tomohiro Yamazaki (University of California, Berkeley)

“Search for supersymmetric partners of the top quark with leptonic signatures”

### Theoretical Nuclear Physics

Tokuro Fukui (RIKEN Nishina Center)

“Development of first-principles shell-model calculations via proper treatment of three-body force”

Yuki Fujimoto (Department of Physics, Graduate School of Science, The University of Tokyo)

“Mapping neutron star data to the equation of state using a deep neural network”

Koichi Murase (Yukawa Institute for Theoretical Physics, Kyoto University)

“Causal hydrodynamic fluctuations in non-static and inhomogeneous backgrounds”

### Experimental Nuclear Physics

TANAKA, Junki (Riken Nishina Center)

“Formation of α clusters in dilute neutron-rich matter”

HAYAKAWA, Shuhei (Tohoku University)

“Observation of Coulomb-assisted nuclear bound state of Ξ^-^ -^14^N system”

YAMAGA, Takumi (RIKEN)

“Observation of a $\overline{\mathrm{K}}$NN bound state in the ^3^He(K^-^, Λp)n reaction”

### Cosmic Ray and Astrophysics

Koichi Hagino (Department of Physics, Faculty of Science and Engineering, Tokyo University of Science)

“Study on ultrasoft outflows from active galactic nuclei and associated absorption structure in their X-ray spectra”

Naoki Aritomi (Gravitational Wave Project Office, National Astronomical Observatory of Japan)

“Frequency-dependent squeezed vacuum source with filter cavity control using coherent control sidebands for gravitational-wave detectors”

Tomohito Fujita (Waseda Institute for Advanced Study, Waseda University)

“Cosmological roles of gauge fields”

### Beam Physics

Kai Huang (National Institute for Quantum and Radialogical Science and Technology)

“Advanced beam diagnostics with electro-optic effects and its application to laser plasma acceleration”

### Atomic and Molecular Physics, Quantum Electronics, Radiation

Kazuya Fujimoto (Institute for Advanced Research, Nagoya University)

“Research on universal scaling in nonequilibrium fluctuations of cold atoms”

### Plasma

Yohei Kawazura (Frontier Research Institute for Interdisciplinary Sciences, Tohoku University)

“Ion versus electron heating in astrophysical plasma turbulence”

Keisuke Fujii (Department of Mechanical Engineering and Science, Kyoto University)

“Statistical modeling of emission spectra from many-electron atoms and heavy nuclei”

### Magnetism

Mitsuru Akaki (Molecular Photoscience Research Center, Kobe University)

“Study of magnetoelectric effects in pulsed high magnetic fields”

Shintaro Takayoshi (Department of Physics, Konan University)

“Theory regarding ultrafast spintronics driven by laser fields”

Yuta Yamane (Frontier Research Institute for Interdisciplinary Sciences, Tohoku University)

“Theoretical study on the electric response of noncollinear antiferromagnets”

### Semiconductors, Mesoscopic Systems, and Quantum Transport

Kariyado Toshikaze (International Center for Materials Nanoarchitectonics (MANA), National Institute for Materials Science)

“Theoretical research on exploring and analyzing novel Dirac systems”

Kenta Takeda (Center for Emergent Matter Science, RIKEN)

“Research on creating silicon quanutum bits and their multiplication”

Tsuneya Yoshida (Department of Physics, University of Tsukuba)

“Research on strongly correlated topological phenomena induced by non-Hermiticity”

### Optical Properties of Condensed Matter

Natsuki Kanda (LASOR, Institute for Solid State Physics, The University of Tokyo)

“Terahertz response and optical control of metamaterials, antiferromagnets, and Dirac semimetals”

Takeshi Suzuki (LASOR, Institute for Solid State Physics, The University of Tokyo)

“Ultrafast spectroscopy on exciton physics”

### Metal Physics (liquid metals, quasicrystals), Low Temperature Physics (Ultralow Temperatures, Superconductivity, Density waves)

Manabu Tsujimoto (National Institute of Advanced Industrial Science and Technology (AIST))

“Terahertz radiation from the intrinsic Josephson junctions of single crystalline high temperature superconductors”

Nayuta Takemori (Research Institute of Interdisciplinary Science, Okayama University)

“Theoretical studies on electron correlation and superconducting states peculiar to quasiperiodic systems”

### Molecular solids

Tetsuya Furukawa (Institute for Materials Research, Tohoku University)

“Quantum criticality and spin liquid states in the vicinity of Mott transitions in quasi-two-dimensional organic conductors”

### Strongly Correlated Electron Systems

Kosuke Karube (RIKEN Center for Emergent Matter Science)

“Study of stability of magnetic skyrmions and exploration of new materials”

Takashi Kurumaji (Department of Advanced Materials Science, the University of Tokyo)

“Discovery of new skyrmion-hosting materials and the effect of centrosymmetry of lattices”

Yasuyuki Shimura (Department of Quantum Matter, Graduate School of Advanced Science and Engineering、Hiroshima University)

“Correlations of hidden degrees of freedom in 4f electron systems by magnetic field responses under very low temperature”

Masamichi Nakajima (Department of Physics, Osaka University)

“Study of electronic structures in iron-based superconductors by optical spectroscopy”

### Surfaces and Interfaces, Crystal growth

Jo Onoda (Department of Physics, University of Alberta)

“Atomic-scale chemical identification and structural analysis by atomic force microscopy”

Koichiro Yamakawa (Advanced Science Research Center, Sector of Nuclear Science Research, Japan Atomic Energy Agency)

“Study of vibrational and nuclear-spin dynamics of molecules with use of in situ terahertz and infrared spectroscopy”

### Dielectrics, Ferroelectricity, Lattice Defects and Nanostructures, Phononic Properties, and X-ray and Particle Beams

Daisuke Morikawa (Institute of Multidisciplinary Research for Advanced Materials, Tohoku University)

“Local structure analysis applied for an interface and an in-situ experiment using convergent-beam electron diffraction”

### Fundamental Theory of Condensed Matter Physics, Statistical Mechanics, Fluid Dynamics, Applied Mathematics, Socio- and Econophysics

Tatsuhiko Shirai (Department of Communications and Computer Engineering, School of Fundamental Science and Engineering, Waseda University)

“Theoretical study on a steady state of periodically driven open quantum systems”

Hidemaro Suwa (Department of Physics, Graduate School of Science, The University of Tokyo)

“Development of efficient Monte Carlo methods for many-body problems”

Satoshi Takada (Department of Mechanical Systems Engineering, Tokyo University of Agriculture and Technology)

“Theoretical study on rheological effects of particle softness for granular gas and suspension”

Hiroyoshi Nakano (Faculty of Science and Technology, Keio University)

“Continuous symmetry breaking and long-range order in nonequilibrium systems under steady shear flow”

### Soft Matter Physics・Chemical Physics・Biophysics

Shunto Arai (University of Tokyo)

“Control of crystallization in soft matter and its application to electronics”

Yuji Sasaki (Hokkaido University)

“Control of topological defects in liquid crystals using self-organization”

Shunsuke Shimobayashi (Princeton University)

“Structure and dynamics in intracellular molecular assembly induced by liquid-liquid phase separation”

## Report on the 47th AAPPS Video Council Meeting by AAPPS

The 47th Council Meeting of the Association of Asia Pacific Physical Societies (AAPPS) was held online from 2:00 p.m. to 5:47 p.m. (UTC+9h) on December 18, 2021, via a Zoom session hosted by the Asia Pacific Center for Theoretical Physics (APCTP). The participants were Jun’ichi Yokoyama (president), Hyoung Joon Choi (vice president), Nobuko Naka (secretary), Keun-Young Kim (treasurer), Gui-Lu Long (ex officio member as a former president), and council members Tao Xiang (the Chinese Physical Society, Beijing), Xiu-dong Sun (the Chinese Physical Society, Beijing), Ruiqin Zhang (the Physical Society of Hong Kong), Mio Murao (the Physical Society of Japan (JPS)), Akira Yamada (the Japan Society of Applied Physics (JSAP)), Woo-Sung Jung (the Korean Physical Society (KPS)), Kurunathan Ratnavelu (Malaysian Institute of Physics), Rajadeep Singh Rawat (Institute of Physics Singapore), Fu-Jen Kao (the Physical Society located in Taipei), Meng-Fan Luo (the Physical Society located in Taipei), and Nguyen Quang Liem (Vietnam Physical Society). Present as observers were Yunkyu Bang (president of APCTP), Je-Geun Park (chair of the Division of Condensed Matter Physics (DCMP)), Youngah Park (chair of the Women-in-Physics Working Group of AAPPS), Eunjeong Lee (AAPPS liaison officer), Dayoung Yang (AAPPS editorial staff), and So-Jin Park (AAPPS liaison staff member). Council member Jodie Bradby (Australian Institute of Physics (AIP)) was absent.

(1) Secretary Naka reported the presence of 16 out of 17 council members (including the ex officio member), and the quorum was declared as fulfilled. Any addition or correction to the proposed minutes of the 46th Council Meeting should be reported to the secretary.

(2) President Yokoyama opened the 47th Council Meeting and welcomed the participants. He explained that this council meeting was originally planned to be held in Hong Kong but the Covid-19 situation did not make it possible. He also mentioned that the Physical Society of Hong Kong has again agreed to postpone hosting a face-to-face meeting, which will hopefully take place before our term ends. The agenda was adopted as prepared by the president.

(3) Yunkyu Bang, the president of APCTP, reported on the progress of activities with AAPPS. As a brief introduction to the history of cooperation between APCTP and APCTP-AAPPS, he mentioned the establishment of the center in 1996 and the relationship between APCTP and AAPPS, which is becoming more intimate. The center consists of 17 entities and 34 theoretical institutes. The countries/regions of these members partly overlap with those of 18 member societies of AAPPS. To advance physics in the Asia Pacific region, the center serves as a hub for academic activities. The support runs for in-house research including 10 Junior Research Groups, 12 Young Scientist Training Programs, and 3 Senior Advisory Groups led by distinguished scholars. There are approximately 50 in-house researchers at APCTP. Furthermore, science diplomacy has been conducted in cooperation with AAPPS, the Asia-Pacific Economic Cooperation (APEC), the International Union of Pure and Applied Physics (IUPAP), and the American Association for the Advancement of Science (AAAS). APCTP’s budget has currently grown to $5 million USD per year. Bang also explained some of the cooperative activities conducted with AAPPS after APCTP’s headquarters moved to POSTECH in 2016. APCTP supports AAPPS in three different categories: $72,000 USD for AAPPS Council and Ordinary General Meetings (OGM), $85,000 USD for APPC9, 10, 13, and 14, and $149,450 USD+53,380 EUR for the *AAPPS Bulletin* (*AB*). During the last three years, starting from 2018, the editorial office has been trying to bring *AB* to a premier level in order to become the major scientific journal in the Asia Pacific region.

Since 2017, 30 million KRW per year has been provided in order to vitalize the division structure in AAPPS. Particularly this year, APCTP supported the inauguration conference of the Division of Condensed Matter Physics (DCMP), which was held in December 2021 with great success. Each of the four divisions receives sponsorship from APCTP to organize conferences and workshops. Bang showed a list indicating that 40–70 total pages were published in each issue of *AB* from 2011 to 2021. Six issues of *AB* are published each year. This year, over 8000 hard copies were distributed to member societies, related institutions, and organizations.

Subsequently, Bang summarized issues to be discussed and finalized: *AB*’s publication contract with Springer Nature and the honorarium for the invited articles.

Starting from 2018, professional management of processing and circulating *AB* has increased the visibility and quality of *AB*. Cooperation with Springer Nature began in January 2021, and Springer Nature has provided various services, such as reporting and assessing the journal’s performance, highlighting the journal content for promotions, and providing an article submission and review system. *AB* has been benchmarked best practices in journal indexing from Springer Nature, which has a lot of experience.

According to Clause 9.2.d, the number of articles to be aimed for each year is specified. An email from the official editors of Springer Nature reconfirmed that it indicates not mandatory but target numbers. If we publish a smaller number of articles than the target, it can put us at a disadvantage in the indexing evaluation.

The initial contract said that the organization will cover the Article Processing Charge (APCs) for up to 20 articles in each year of 2021–2023 and up to 26 articles in each year of 2024 and 2025. After friendly negotiations, APCTP chose to cover the APCs for up to 40 accepted articles (invited Research & Review articles only) from 2022 to 2025.

It had been unclear whether AAPPS is allowed to use the content of published articles in print or in an electronic form. According to the original contract, AAPPS and APCTP have a right to reproduce articles in print and in an electronic form, which applies only to News & Commentaries and excludes peer-reviewed articles. However, the editorial office has received a formal letter that Springer Nature agrees to print all the materials for association members during the period of the current agreement.

The amendment to the contract will be signed by three bodies, i.e., the publisher (represented by the Editorial Director), AAPPS (represented by President Jun’ichi Yokoyama), and APCTP (represented by President Yunkyu Bang).

The concern about the honorarium paid by APCTP for writing articles in *AB* is not yet settled. Currently, $1000 USD/paper is paid for invited authors and $2000 USD for scholars of Nobel laureates or of a similar rank. A concern was raised by some Japanese authors and editorial board members that the amount might be too high for researchers. The center has a technical problem with changing the payment style during a financial year and will pay the same amount at least during this year. After some discussion, it was agreed to reduce the honorarium to $500 USD/paper for a standard invited article, starting from next year [see item (4)].

The ceremony for the CN Yang Award 2021 was held in October and the next year’s timeline was already announced. There was a revision of the rule for nomination; each member entity of APCTP can make up to three nominations every year. Issues to be discussed are the prize money and the venue of the ceremony. $1000 USD shall be provided if the awardee submits an article to *AB*. However, only two awardees have received the prize money since 2019. APCTP is willing to increase the prize money to $3000 USD in order to encourage awardees to write articles more punctually. Another possibility is to change the venue of the award ceremony. APPC conference sites are suited to honor awardees every 3 year. However, during the other 2 years, awardees are celebrated at the APCTP alumni conference site, which is a local and small-scale conference and might discourage the winners. Bang suggested holding a ceremony at an annual meeting site of a member society in rotation or at an annual council meeting site.

Bang then recognized the cooperation and support received from AAPPS.

Gui-Lu Long asked about the meaning of the phrase “benchmarked best” and Eunjeong Lee clarified the meaning. Long stated that the prize money should be given to awardees regardless of writing an article or not, and that some additional money can be provided to invited authors. Bang responded that he basically agrees with Long but the problem is mainly because the center, which is supported by the Korean government, has technical and administrative difficulties to simply give money to awardees. Long commented that editors may reject an article if it is not well written, so the award money and writing should be separated. Tao Xiang proposed asking awardees to write a commentary explaining why they received the prizes instead of a full article. Long and Bang agreed with this reasonable solution. Long added that editors do not reject such short articles. Yokoyama asked Bang to consider this suggestion under boundary conditions.

Kurunathan Ratnavelu greatly appreciated all the funding for many conferences besides APPCs from APCTP over 25 years. As a member of the Malaysian Institute of Physics, he hopes to send a special contribution to APCTP. Bang mentioned that they planned to celebrate APCTP’s 25th anniversary, which was not realized due to the spread of Covid-19 last year.

Yokoyama commented that the budget reaching $5 million USD and the increased rate of support during the past theree years look significant. Yokoyama asked whether the financial support to APCTP is in accordance with the Korean government’s typical disbursement of their science budget or whether APCTP is receiving funding at a higher rate. Bang answered that APCTP’s administration office worked well together with the government.

(4) Long, the editor-in-chief of the *AAPPS Bulletin* (*AB*) reported on the current status of *AB*. Cooperation with Springer Nature started in 2021, and there are presently three forms, i.e., two online forms (the same as before and on the Springer Nature web page) and one printed form. There is a link to the Springer Nature web page through the *AB* web page. The Chinese Physical Society, Beijing, JPS, KPS, and the Physical Society located in Taipei have been providing contributions ($20,000 USD in total) to *AB*. JSAP has moved to support access to *AB* for the general use of AAPPS, this year. Another $29,200 USD was provided this year by APCTP for printing and editorial board meetings. There are two categories for cooperate members, which generates a total income of $17,250 USD. The balance is 147,141 KRW or $124,281 USD. Two meetings of all editors are held in June and December each year. Their term-ending dates are divided into three respective times in May 2023, 2024, and 2025.

In cooperation with Springer Nature, citations are improving (11 articles cited among 20 according to Google Scholar) and the performance is getting better. The number of articles published this year will exceed the target number of 30. Although up to 40 articles are to be covered by APCTP, *AB* is trying to run in a self-sustaining way in the future. Long asked the council members to promote *AB* whenever and wherever possible.

APCTP currently provides $1000 USD for the first author of an invited article. Some Japanese authors expressed the concern that the amount may be too high. For reference, an honorarium for an article in the *Bulletin* of JPS, the Chinese Physical Society, the Physical Society located in Taipei, and that for an invited review in European journals were surveyed. Considering the length of the articles and to avoid any conflict with some member societies, Yokoyama proposed $500 USD. The proposal was approved by the council members.

(5) Yokoyama suggested discussing the two issues regarding the CN Yang Award, as pointed out by Bang. That is, whether we should increase the prize money and/or change the venue for the ceremony. Bang expressed his concern that according to the preceding discussions, they will face the same problem as the honorarium if the prize money is increased to $3000 USD. He suggested just passing $1000 USD without obligation or asking for a short commentary of 1–2 page(s) on the winner’s achievements. Long added that an invited review article is also welcome.

Woo-Sung Jung suggested postponing the discussion on the venue in 2023 until August 2022, because the time of the council meeting can be set flexibly. If we would suggest for the venue to be rotated, then it should be discussed in the next OGM as each member society should invite awardees. Yokoyama agreed that this is not an immediate issue. Long proposed to hold ceremonies at annual meetings of respective awardee’s societies attended by a representative of APCTP or of AAPPS.

Fu-Jen Kao clarified the number of nominations through members of APCTP, AAPPS and APCT overlap in some countries/regions but not in other countries/regions. In both cases, six nominations are possible. Yokoyama explained that the underlining idea of having APCTP recommend nominees is that the CN Yang Award is a joint award of AAPPS and APCTP, and both entities should be treated equally. Bang stated that there is no problem with increasing the number of nominees. Kao gave one more comment that more efficient logistics of the selection procedure should be prepared if an increased number of nominations are expected in the future.

(6) Vice President Choi proposed a discussion on the legal entity issue. He explained that we discussed having a legal entity of AAPPS in the council meeting one year ago. The current bank account is a personal one in the name of the treasurer. The legal entity should be independent and should make its own decisions. The board of trustees should get together at least once a year face-to-face. The next step is to prepare an executive plan by the next council meeting or by the council meeting after the next one.

Yokoyama asked how long it would take, after making a clear plan with a list of pros and cons. Jung answered that a couple of months will be necessary for administrative preparation for the application and that the Korean government should review the entity for approval. Yokoyama voiced that we need real people to join the board of trustees, and he asked if the KPS could help us with this process. Jung answered yes and responded that the application itself would not be a serious issue.

As the present account is in the name of the treasurer, we would have to move the assets to the legal entity’s account. However, the transfer of the money may be a complicated process taking more time and would need support from a lawyer. The point is that the transfer of money between nonprofit organizations might cause a taxation problem. Akira Yamada explained that JSAP is a nonprofit organization categorized as a public interest incorporated association and now suffers from many troubles. Jung pointed out that AAPPS should be a nonprofit entity because for-profit organizations in Korea pay a large amount of tax. A similar problem arose when we received a donation from the USA because APCTP is also a nonprofit organization. Yokoyama suggested taking more time and carefully investigating more in the proposed direction.

Jung stated that the AAPPS Bulletin (*AB*) money is kept under the APCTP account. To take responsibility for the money, which is not the income of APCTP, is also a burden for APCTP. Choi voiced that the difficulties may grow as funds increase, if we do not think about it now. Bang asked Jung if he consulted with Prof. Kikuchi of the Division of Plasma Physics. Jung responded that he did not do so because Japanese laws are different from Korean laws. Yokoyama summarized that having a legal entity should be realized somehow sooner or later. Choi added that the final approval should be made at the OGM in August 2022, after a decision is made in the next council meeting.

Rajdeep Singh Rawat asked about the transfer procedure of membership fees from a member society. It was confirmed that it is safer to add the treasurer’s name together with AAPPS in the case of a bank transfer.

As the chair of the DCMP, Je-Geun Park expressed his thanks to Presidents Bang and Yokoyama, for their general advice and financial support. He explained that DCMP is a young division surviving this difficult time. At the first meeting on November 18, 2019, the participants agreed to establish a new division with the support of more than 25 stakeholders. The AAPPS Council approved the proposal on December 10, 2020, and the division launched on January 1, 2021. There have been five founding societies and 10 executive committee members. The mission of DCMP is to strengthen academic networking with the aim of playing an active role in shaping the future of condensed matter physics.

The membership to DCMP is handled automatically by logging into the web page after creating an account. The division publishes newsletters twice times a year. The domain is for exchanging information of current activities of each society and for encouraging scientific presentations of the results in a short article format. The Asia-Pacific Conference on Condensed Matter Physics (AC^2^MP) went well with 372 participants from 14 societies, 51 speakers, and 33 posters. The key elements of the event were the tutorial session and the special session with publishers.

Rawat confirmed that the membership fee will be free for at least the first 5 years. J.-G. Park explained that online application through the website automatically goes to the vice chair. He collects and accumulates applicants’ information for a month and sends those names to each society for approval. The societies review the applicants and give their approval.

Choi wondered if approval is made by the five member societies, and if the individual applicant is supposed to belong to one of the five member societies. J.-G. Park answered that they had a discussion on the extension of membership at the second exco meeting. On October 2, 2021, a call to join DCMP was made to each member society of AAPPS by email. The division has received interest from Singapore and Australia. Those two countries are expected to join soon as new member societies of DCMP. Also, the involvement of individual members from a non-member society of AAPPS will become possible in the next year.

(8) Ratnavelu reported on COMDATA2021, which incorporated the 1st South-East Asia Workshop on Computational Physics & Data Analytics in Sciences (CPDAS 2021). COMDATA2021 was attended by 90 participants from 14 countries. As described in the report sent by email, the plenary talks covered broad areas of physics. He explained that they should have a strong section of computational physics in the coming APPC and it will lay the basis for the Division of Computational Physics in AAPPS. However, Ratnavelu wondered if it could be a topical group rather than a division. Ruiqin Zhang and others agreed with his opinions.

(9) Choi reported on the current status of preparations for APPC15. The plenary sessions are scheduled for each morning. A call for nomination of plenary talks was sent by email, whose deadline of January 11, 2022, is not a hard one. The International Program Committee will review the nominations and select speakers considering gender, subject balance, and so on. A special session titled Global Physics Summit is planned on August 22, 2022, to be attended by presidents of AAPPS members societies, IUPAP, the European Physical Society (EPS), the American Physical Society, and APCTP. This special forum hosted by the KPS on the 70th anniversary of KPS is intended to share and discuss visions and perspectives for global and regional collaborations in promoting physics research and education.

The format of APPC15 (i.e., on-site, online, or hybrid) will be decided in March or April 2022. The registration fee is slightly increased to $380 USD for regular participants, while the student fee remains at $250 USD. The members of the International Advisory Committee, International Organizing Committee, and Local Organizing Committee were introduced. The International Program Committee consists of currently 160 members but will soon reach 200.

Rawat asked if the registration fee will be changed when the conference format is changed. Choi responded that the fee will be slightly reduced in case of a fully online format. Rawat wondered if a single fee is applied both for offline and online participants in the hybrid case. Choi answered that it is not exactly decided yet but probably a 10 % discount will be applied for fully online participants.

Choi added that if any council member would like to recommend additional members in the International Program Committee from his/her society, it is welcome. Yokoyama suggested including everyone in the preparation group of the Division of Particles and Fields. Choi answered that a number of them will be invited and asked Keun-Young Kim to invite more. Choi will request committee members of each subject to invite scholars from member societies that are not yet included, i.e., Singapore, Hong Kong, Australia, and New Zealand.

Rawat clarified if the poster session slots are sufficient to take all applications. Choi responded that they will adjust the times depending on the number of posters.

(10) Treasurer K.-Y. Kim gave a brief report on the financial status of AAPPS. The summary of the AAPPS account during 2020–2021 was presented and the balance of 74,036,512 KRW or $61,690 USD, in addition to Leo Koguan Foundation’s $36,500 USD, (with $10,000 USD excluded for *AB*) was reported. The account statements include membership fees and support from APCTP of $468,360 USD. The Division of Astrophysics, Cosmology and Gravitation supported the domain fee of $113 USD.

(11) Yokoyama explained that we organized the Asia-Pacific Physical Societies’ Forum on November 23, 2021. He expressed thanks to Naka for her work regarding arrangements. The meeting was very successful with contributions from many AAPPS member societies. The presence of Nepal in AAPPS has not been strong, historically. However, during the forum, the president of the Nepal Physical Society introduced their activities, including an online international conference scheduled for 2022, and asked for an endorsement from AAPPS. The AAPPS logo has now been attached to the conference website. Long, Choi, and Yokoyama will join the conference as keynote speakers. Yokoyama furthermore proposed supporting the conference financially. If our presence becomes more prominent in Nepal, it is advantageous for AAPPS. As some countries have difficulties in sending money abroad, Yokoyama suggested supporting Nepal through their events by compensating Nepal through their unpaid AAPPS membership fees. Rawat supported the idea and K.-Y. Kim stated that he had already agreed. Kao supported it as well. Yokoyama will tell them to “pass” something equivalent to $2000 USD to Nepal together with encouragement to organize more AAPPS-related events in the future.

Thanks to the efforts of DCMP, India has now become an active member of AAPPS. Yokoyama asked about the status of Indonesia, Mongolia, and New Zealand.

(12) AAPPS sent an invitation to Uzbekistan to join the Asia-Pacific Physical Societies’ Forum. Subsequently, Uzbekistan sent a representative to deliver a talk as a guest speaker and has showed an intent to join AAPPS. Currently, our newest member is Kazakhstan. There was some negotiation with EPS because former Soviet Union countries were regarded to be in the EPS region at that time. The former president Long and former secretary Yokoyama reached an agreement with EPS that these countries can join both EPS and AAPPS. Yokoyama showed a letter from the president of the Council of Uzbekistan Physicists. This is a newly established society representing physicists in Uzbekistan, with approximately 500 members. Their initial income will be very limited, but they have already established divisions and are publishing three journals.

Yokoyama asked for the opinions of the council members for Uzbekistan to join AAPPS, with a remark that they further asked us to waive the membership fees for the first 3 years. Yokoyama proposed providing financial support of $1,500 USD for paying membership fees instead of waivers. Xiang, Rawat, and Ratnavelu expressed their support and other council members also agreed with the idea.

(13) Yokoyama stated that our term will end on December 31, 2022. He confirmed that all the council members agree to have the next OGM at the occasion of APPC15, attended by the presidents of member societies. He explained the constitution and bylaws describing the rule for the election of the council members. We must fix the size of the next council six months before the OGM. Clause 5.1 of the constitution sets the maximum number of 15 (+1+1), where (1+1) means a new secretary and a new treasurer, to be nominated by the next president, in case they are nominated from outside the new council.

Yokoyama first proposed following the current size of 15+1+1. However, the number of our member societies is increasing and there is another option to change the constitution in an extraordinary general meeting. Luo and K.-Y. Kim suggested having more council members in the future. Since this requires a change of the constitution, which can only be done in the general meeting instead of a council meeting, Yokoyama suggested to limit the number of council members from each society to two so that more societies could send a council member. Although currently the Chinese Physical Society, Beijing, JPS, and KPS are sending three council members, none of them have three different opinions, and furthermore, quite often, some of the members are absent. Nguyen Quang Liem voiced that we should encourage small new societies to join the council. For this purpose, we could create a limit where one member society could only send only one council member. Long opposed and stated that we should not change the current format because each member society has only one vote at the OGM for democracy. The AAPPS council members are elected at the OGM, and the council is a working body, which is more concerned with efficiency.

Subsequently, Long proposed creating a new position of associate council members without voting rights, so that more member societies can join discussions and express their opinions in council meetings, just like in the previous council meeting in Xi’an where representatives of all the member societies were invited in addition to the council members. Yokoyama summarized that we will keep the size of the council as described in the constitution, and we will invite one representative as an associate council member from each society that presently does not send council members.

(14) Yokoyama explained that we will announce the venue for APPC16 at the time of APPC15. Rawat informed that there was a discussion in the council meeting of the Institute of Physics Singapore (Singapore hosted the first APPC in 1983). Yokoyama asked about the necessary time for each member society to prepare for nomination. Ratnavelu suggested giving three months. The council members agreed to set the deadline for nomination at the end of April 2022.

Long proposed that the chair of the CN Yang Award selection committee should be decided much earlier, and recommended Rawat for the next chair of the selection committee. Rawat accepted the position after asking for advice from Kao, the chair of the 2021 selection committee. The council unanimously agreed to appoint Rawat as the next chair of the selection committee.

(15) Yokoyama announced that the next council meeting will be organized online sometime in May 2022 and that any updates on APPC will be circulated by email.

